# Mentoring future Kenyan oncology researchers

**DOI:** 10.1186/1750-9378-8-40

**Published:** 2013-10-07

**Authors:** Ann Moormann, Jodi Skiles, Emmanuel Koros, Fredrick Chite Asirwa, Naftali Busakhala, Patrick Loehrer

**Affiliations:** 1 University of Massachusetts Medical School, Biotech II, 373 Plantation Street, Suite 318, Worcester, MA 01605, USA; 2 Indiana University School of Medicine, 705 Riley Hospital Drive, ROC Room 4340, Indianapolis, IN 46268, USA; 3 College of Health Sciences, School of Medicine, Department of Medicine, Moi University, Eldoret, Kenya; 4 Academic Model Providing Access to Healthcare (AMPATH), Eldoret, Kenya

**Keywords:** Cancer, Kenya, Grant writing workshop, Provocative research questions

## Abstract

This is a summary of the 1^st^ Academic Model Providing Access to Healthcare (AMPATH) Oncology Institute research grant writing workshop organized in collaboration with the Kenya Medical Research Institute (KEMRI) and held in Kisumu, Kenya from January 16^th^ to 18^th^, 2013. The goal of this meeting was to mentor future Kenyan scientists and prioritize research topics that would lead to improved cancer care and survival for the citizens of Kenya.

## Introduction

Medical Scientists drawn from the Academic Model Providing Access to Healthcare (AMPATH) Consortium collaborated with the Kenya Medical Research Institute (KEMRI) in Kisumu to host the first United States, National Cancer Institue (NCI) sponsored oncology-focused grant writing workshop. The goal of this workshop was to mentor health care researchers and to prioritize research topics that could lead to improved cancer care and survival for the citizens of Kenya. The spectrum of cancer research included prevention, early detection and screening as well as palliative care. Twenty mentees out of 34 applicants were selected through a competitive process in which submitted applications were reviewed based on research interests, how those interests could improve cancer care and outcomes, and how this training would facilitate their careers in medical research. Ideas for research proposals were stimulated by a series of Provocative Questions given to applicants prior to the meeting (Appendix 1).

Kenya, like much of the developing world, is rapidly undergoing an ‘epidemiologic transition’ from a health scene dominated by infectious diseases to one in which the chronic diseases such as cancer are becoming major causes of death and disability. Under these circumstances, applying science to the management and control of cancer has become as relevant to Kenya as it is in the United States and other countries. Cancer research in Kenya, whose population is the most genetically diverse in the world [1], will catalyze the discovery of new genes of importance in the fight against cancer, new genomic predictors of cancer, and new genetic variants that predict response to therapy. AMPATH Oncology Institute (AOI) activities aim to directly contribute to advances in cancer care and accelerate discoveries in the biology and treatment of cancer in Kenya. One of the most important components of this mission is training Kenyan scientists and strengthening the collaborative research environment.

### The AMPATH consortium

AMPATH stands for the Academic Model for Providing Access to Healthcare (AMPATH: http://www.ampathkenya.org/). AMPATH opened its first clinics in 2001, at a time in which the AIDS epidemic dominated the health landscape of sub-Saharan Africa. The AMPATH model is a partnership between Moi University School of Medicine (MUSOM), Moi Teaching and Referral Hospital (MTRH, Kenya’s second national referral hospital), and a consortium of U.S. and Canadian medical schools led by Indiana University School of Medicine. North American AMPATH consortium partners also include the Alpert Medical School of Brown University, Hubert-Yeargan Center for Global Health at Duke University Medical Center, Lehigh Valley Health Network of Pennsylvania, Eck Institute for Global Health at Notre Dame University, Providence Portland Medical Center, Purdue University, University of Massachusetts Medical School, University of Utah School of Medicine and University of Toronto Faculty of Medicine. Institutions participating in the AMPATH Consortium engage in student and faculty exchange, clinical care, training, and research, all essential for successfully addressing the short- and long-term challenges of global health. While training and research are critically important to the program, AMPATH has always been determined to “lead with care.” Care is not only the most pressing obligation when faced with the needs of an under-served population, it is also the foundation upon which training and research is conducted.

Some of our newer programs focus on chronic diseases such as cancer. Within this context, AMPATH is creating a Center of Excellence in Cancer Care with the naming of the AMPATH Oncology Institute (AOI). The goal of AOI is to be the premier cancer program in Sub-Saharan Africa, noted for excellence in cancer research, prevention, treatment and palliative care. And in order to meet this need, specialized training in oncology and oncology-related research is imperative.

### AOI research grant writing workshop

A three-day symposium was conducted from January 16^th^ to 18^th^, 2013 in Kisumu, Kenya which included doctors, nurses and other health-allied scientists affiliated with the Hemato-Oncology Department at the MTRH and AMPATH Oncology, located in Eldoret. Faculty mentors from the United States and Kenya dedicated three days to teaching participants about writing grant proposals for conducting research in oncology.

The most prevalent cancers seen at AOI are listed in Table 1. In 2012, doctors at AOI saw over 800 newly diagnosed cancer patients resulting in over 4000 cumulative patient visits during the course of the year for oncology-related matters and the administration of over 3500 cycles of chemotherapy. Some of these appointments consisted of follow-up care and monitoring that did not require treatment, however the increasing number of diagnosed patients endorses the need for an equally increasing number of trained oncologists and support staff. Screening for breast and cervical cancer has also become an important service to the community. MTRH-AMPATH alone screened over 7000 women in 2012. Recognizing cancer as a priority health concern in Kenya, the IU Simon Cancer Center (IUSCC) and the AMPATH Program have been actively pursuing resources to respond. We have found that some patients are unable to complete the full course of treatment due to late stage at time of diagnosis resulting in immediate referral to palliative care. Financial burden placed on the family can also result in abandonment of care. Therefore socio-economic issues leading to poor compliance are also important topics for research. The overall focus of the partnership is to develop a sustainable and comprehensive academic clinical care program that will serve the citizens of western Kenya. MTRH currently has five formally trained medical Oncologists, six Pathologists and two radiotherapy machines intended to serve the entire country. Enhancing the clinical capacity will also establish a solid foundation upon which important research questions can be investigated.

**Table 1:** Prevalent cancers at AOI 2001-2011

**Diagnosis**	**No. of cases**
Kaposi’s sarcoma	1161
Cancer of esophagus	998
Cancer of cervix	856
Leukemia	789
Breast cancer	748
NHL/Burkitt lymphoma	748
Prostate cancer	482

The workshop was chaired by Drs. Ann Moormann and Jodi Skiles. Presenters at the workshop included Prof. Thomas Inui (Indiana University School of Medicine, USA), who provided an overview of grant writing and how important it is to learn how to convey your research ideas, objectives and desired outcomes. Clearly explaining how research can translate into better care and survival for patients is an important aspect of successfully funded grant proposals.

Dr. Stephen Munga (KEMRI-Centre for Global Health Research, Kenya) followed with a talk on “Conducting Health Services Research in Low Income Countries.” He stressed the importance of understanding local and regional determinants of health, knowledge of ongoing health research efforts – including how social factors such as family, community, and the central government play a role in contributing to health and health behaviors. He further emphasized that limited resources, inadequate infrastructure, the effects of “brain drain” and limited communication between researchers and policy makers pose great challenges to researchers in developing countries.

Prof. Patrick Loehrer (Director of the IUSCC and the AOI), covered the basic tenets of writing a clinical trial. Another important aspect of entering into the research arena was presented by Prof. Anthony Mega (Alpert Medical School at Brown University, USA) who delivered a lecture titled: “Mentorship: the art of choosing, using and becoming a mentor.” Mega said that a mentor should be a role model, an advisor, an encourager, a resource person, a counselor, sponsor and a friend. They ought to inspire, support and invest in their mentees. Research is often a team endeavor whereby scientists with different strengths bring their expertise to the table.

Another key topic of interest “Integrating Translational Research into Clinical Research” was introduced by Prof. Alan Rosmarin (University of Massachusetts Medical School, USA). He opened the discussion by posing a question to participants about what barriers exist to implementing translational research in Kenya. The participants cited limited availability of laboratory technology available, academic silos within the government, and a lack of access to care for all citizens.

While delivering a talk on Manuscript Writing, Prof. Ann Moormann (University of Massachusetts Medical School, USA) emphasized that upon getting results, the researcher must strive to tell their target audience what those results mean by interpreting the data and explaining how those results add to what is already known about the topic. Writing the manuscript should take high priority because it is the end product of research efforts and adds to the body of knowledge within the medical community. Another salient point highlighted was that publication costs for scientists in resource-limited settings may be waived or greatly reduced by many journals. She also mentioned the value of publishing in open-access journals which are available to your colleagues without a subscription fee.

Other presenters included Prof. Edwin Were (Moi University, Kenya), who delivered a lecture on ethical considerations for conducting research in resource-constrained settings and Dr. Christine Wasunna, (KEMRI, Kenya) who gave a talk on cultural considerations in community-based cancer research in Kenya. David Mulama, (KEMRI, Kenya), presented a brief tutorial on data management and analysis, mentioning statistical programs that are often available as open source software. Prof. Greg Gramelspacher (Indiana University School of Medicine, USA) shared his experience on palliative care research in low and middle-income countries. Providing comfort and relieving suffering for those diagnosed with cancer is a critically important aspect of cancer care.

The morning presentations were followed by afternoon small group sessions where four to five mentees worked directly with two to three mentors. These sessions allowed the mentees to ask more specific questions about their research proposals and seek feedback from their peers. The evenings were free time for the mentees to work on their project presentations.

### The peer-review process

The workshop format included short (10–15 minute) individual research proposal presentations from each of the mentees. After each participant presented their work, the proposals underwent a peer-review process. The three best proposals were selected by the other participants and given special recognition. This process was to simulate peer-review for grant applications whereby only the best proposals get selected for funding. This also allowed the mentees to understand how they could improve their research proposal by integrating constructive suggestions into their revisions.

The concluding presentation was an inspiring talk by Dr. Kirtika Patel (Moi University, Kenya) who gave a brief overview of her research journey covering the key elements of making the transition from mentee to mentor. She urged the future researchers to exercise patience when writing their proposals, bearing in mind that persistence is a critical key to success.

### Future directions

The long-term goal of this workshop is to continue to strengthen research partnerships between collaborators from various institutions that can be leveraged to apply for grant applications. This is being accomplished by continued mentor-mentee communication via the internet and weekly conference calls, helping to identify requests for grant applications that would be amenable to the situation in Kenya, and ongoing grant writing mentorship.

## Appendix 1

Provocative Research Questions

Breast Cancer

● Is breast cancer in Kenya different from North American patient populations?

● Why do younger women get breast cancer in Sub-Saharan Africa?

● What obstacles to early detection of breast cancer can be overcome in Kenya?

● How effective is standard chemotherapy or hormonal therapy in breast cancer in Kenyan women?

● Are there associated risk factors for breast cancer in women of Kenya?

Cervical Cancer

● What are the Human Papillomavirus (HPV) serotypes for cervical cancer in Kenya?

● What is the impact of antiretrovirals in patients diagnosed with and treated for cervical cancer?

● Is single agent chemotherapy effective in advanced cervical cancer?

● Are there regional differences in incidence and outcome of cervical cancer?

Lymphoma

● What are the most common histologies of non-Hodgkin lymphoma (NHL) in western Kenya?

● How accurate is histologic identification of NHL in Kenya?

● Will improved subclassification of NHL improve outcomes of lymphoma therapy in Kenya?

● What is the safest and most effective treatment for NHL in resource constrained setting?

Palliative % Supportive Care

● What is the scope of pain management in Kenya and what opportunities exist to enhance it?

● Do vitamin (e.g., folate) supplements improve outcome when combined with chemotherapy?

● What are public perception and beliefs of cancer?

● What are obstacles to vaccine therapy in adolescent girls and boys in Kenya?

● Can a culturally appropriate model to end-of-life communication be developed and implemented in western Kenya?

Kaposi Sarcoma

● Why is Kaposi sarcoma (KS) more prevalent in Kenya compared to US?

● Why is severity and extent of KS worse in Kenya?

● Can optimization of dermatologic care improve palliation of KS patients?

● Is there a drug-drug interaction with antiretroviral therapy and chemotherapy in patients with KS?

Pediatric Oncology

● Is acute lymphoblastic leukemia (ALL) different based on cell phenotype or cytogenetics in Kenya patients compared to children in North America (NA)?

● Is Wilm’s tumor in Kenya different compared to NA by protein profiling?

● What obstacles to early detection of leukemia/lymphoma can be overcome in Kenya?

● How effective is standard chemotherapy in ALL?

● Is it feasible to utilize flow cytometry for the diagnosis of leukemia and lymphoma in Kenya?

● What is the impact of antiretroviral therapy in patients diagnosed with NHL/Burkitt’s?

● Are there regional/ethnic differences in incidence and outcome of leukemia?

● What are the most common histologies of pediatric NHL in western Kenya?

● How accurate is histologic identification of pediatric NHL in Kenya?

● What is the safest and most effective treatment for pediatric NHL and ALL in a resource-limited setting?

● What are the factors that contribute to abandonment of care in pediatric oncology patients in Kenya – and are they different than those seen in adults?

● How can pediatric cancers (specifically leukemia, NHL, Wilm’s, and retinoblastoma) be identified and referred earlier for care?

● Can a cost-effective sustainable model for cancer care be established in Kenya?

● Why is there such a discrepancy in survival between pediatric ALL patients in North America and Kenya?

● What are the main causes of mortality in the context of therapy for pediatric malignancies?

Health behavior, health education topics

● Why do many patients with potentially preventable conditions fail to present for cancer screening services within the AMPATH system of care.

● Corollary question: Why do some AOI patients in care for malignancies drop out of care?

Other topics

● What are the Human Papilloma Virus (HPV) serotypes in head and neck cancer in Kenya?

● What is prevalence of smoking and exposure to smoke (homes) in Kenya and association with cancers?

● Can a cost-effective sustainable model for cancer care be established in Kenya?

● What is the impact of economic barriers (perceived or real) in seeking and sustaining cancer care?

● What financing model (i.e. sliding-fee scale) is appropriate in Kenya?

## Competing interests

The authors state no conflict of interests.

## Authors’ contributions

AMM and JLS drafted manuscript, organized meeting and mentored attendees. EK drafted manuscript and attended meeting. FCA and NB drafted manuscript and helped organized meeting. PL drafted manuscript, organized meeting and mentored attendees. All authors approved the final manuscript.
